# Environmental Impact Prediction of a New Tire Vulcanization Activator

**DOI:** 10.1021/acssuschemeng.3c06640

**Published:** 2024-04-10

**Authors:** Thomas Hennequin, Lotte van Vlimmeren, Silvia Mostoni, Francesca Rita Pomilla, Roberto Scotti, Claudia Stauch, Mitchell K. van der Hulst, Mark A. J. Huijbregts, Rosalie van Zelm

**Affiliations:** † Department of Environmental Science, Radboud Institute for Biological and Environmental Sciences, Radboud University, P.O. Box 9010, 6500 GL Nijmegen, The Netherlands; ‡ Department of Material Science, INSTM, University of Milano-Bicocca, Via Roberto Cozzi 55, 20125 Milano, Italy; § Institute for Photonics and Nanotechnologies-CNR, Via Alla Cascata 56/C, Povo, 38123 Trento, Italy; ∥ Fraunhofer Institute for Silicate Research, Neunerpl. 2, 97082 Würzburg, Germany; ⊥ Expertise Group Circularity & Sustainability Impact, TNO, P.O. Box 80015, 3508 TA Utrecht, The Netherlands

**Keywords:** life cycle assessment (LCA), prospective, ex ante, green chemistry, tires, case study

## Abstract

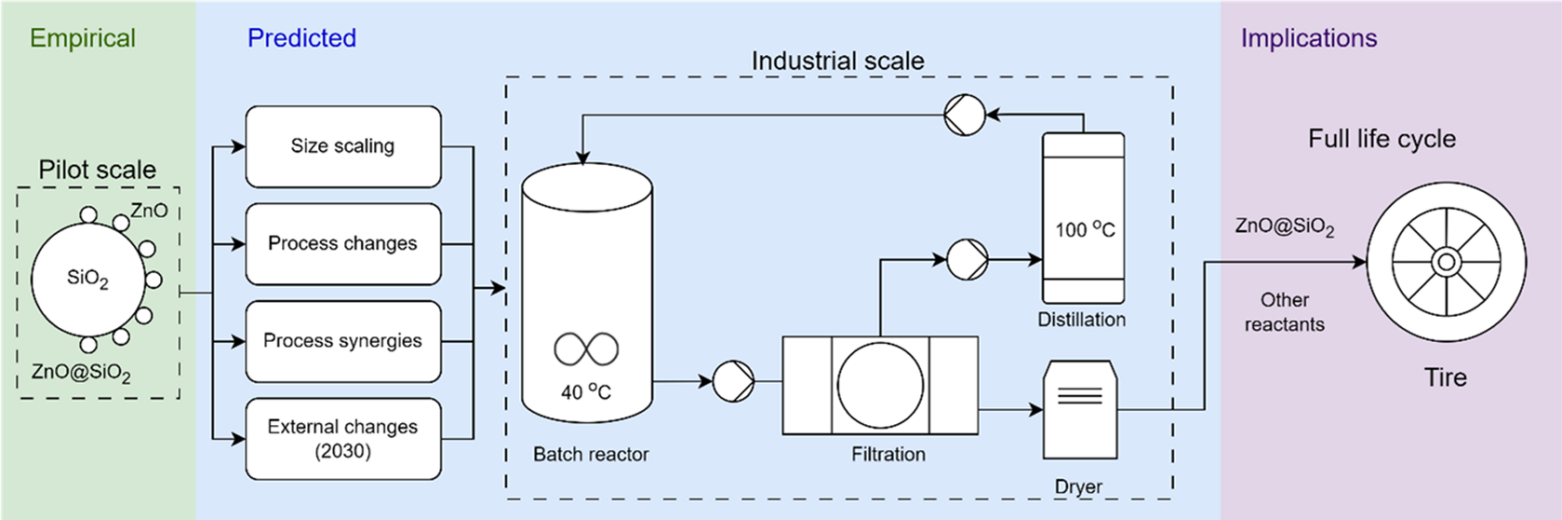

Zinc oxide (ZnO) is the most common curing activator used to manufacture tires. To minimize environmental impacts by decreasing the zinc content and rolling resistance of tires, ZnO nanoparticles (NPs) anchored on SiO_2_ NPs (ZnO@SiO_2_) are currently under development as new activators at the pilot scale. Here, we applied prospective life cycle assessment to predict the impacts on human health, ecosystem quality, and resource scarcity of synthesizing ZnO@SiO_2_ for the production of passenger car tires at an industrial scale. We found that the life cycle impacts of the synthesis are expected to decrease by 89 to 96% between the pilot and industrial scale. The largest contributors to the synthesis of ZnO@SiO_2_ were electricity consumption and waste treatment of the solvent. Using the new activator for tire production led to potential reductions of 9 to 12% in life cycle impacts compared to tires that are currently in use. Those reductions were due to the expected decrease in rolling resistance, leading to lower fuel consumption, which outweighed the additional environmental impacts of the synthesis, as well as the potential decrease in lifetime. Our work highlights an opportunity for manufacturers to mitigate their impacts over the full life cycle of the tire.

## Abstract 2

The industrial scale environmental impacts of synthesizing the new ZnO@SiO_2_ tire vulcanization activator are predicted using prospective life cycle assessment.

## Introduction

Road transportation was responsible for 18% of Europe’s greenhouse gas emissions in 2019.^1^ Environmental life cycle assessments (LCAs) have shown that the fuel consumption of internal combustion engines is the main contributor to passenger cars’ carbon footprint and the majority of other impact categories.^2−4^ The tire is responsible for 20 to 30% of a car fuel consumption, depending on driving conditions.^5,6^ Rolling resistance is the force that resists the motion as it rolls on a surface and is the main characteristic that influences fuel consumption. Therefore, reducing tire rolling resistance within the limits of the safety regulations is a key strategy to reduce the environmental impacts of road transportation.^7,8^

Zinc oxide (ZnO) is globally used as an activator during the vulcanization of tires to improve curing efficiency and plays a role in determining tire characteristics, notably rolling resistance.^9−11^ LCAs have also shown that zinc from tire wear is a large contributor to the impact of tires on marine (71%) and human toxicity (72%)^8^ as well as to the impact of cars on terrestrial toxicity (40%).^7^

Susanna et al.^12,13^ developed a new activator using ZnO nanoparticles (NPs) anchored on silica NPs, namely, ZnO@SiO_2_ (shortened from ZnO-NP@SiO_2_–NP), as an alternative to the microcrystalline ZnO currently used in commercial tires. This new activator can lead to lower ZnO usage and environmental hazards,^14^ a shorter curing time, a higher cross-linking density, and thus improved elastomeric properties.^12^ Those changes could potentially lead to environmental benefits due to the expected decrease in tire rolling resistance and also to a possible increase in environmental burdens in the production phase due to other changes in tire characteristics. However, it is not clear how a change in the activator would play out over the full life cycle of the tire.

As the new activator is not produced at a commercial scale yet, a prospective LCA (pLCA) is needed for a fair comparison against commercially available ZnO. pLCA has been used to predict the future environmental impacts of emerging technologies over their full life cycle.^15−20^ pLCA allows an emerging technology to be modeled at a future, more-developed phase while it is still in early development.^21−25^ Alternative techniques or products can be evaluated to propose a design with lower environmental impacts.^15,26^ Case studies on nanomaterials, aerogels, and graphite showed that environmental impacts decrease when emerging technologies are scaled from laboratory to industrial scale.^15,17,27^

The goal of this study was to predict the environmental impacts of synthesizing the new ZnO@SiO_2_ vulcanization activator at an industrial scale in 2030. pLCA was used to model the manufacturing of the new activator at technology readiness levels (TRLs) 5, 6, and 9 by using the frameworks of van der Hulst et al.^25^ and Piccinno et al.^28^ We investigated the change in environmental impact with increasing TRL, the contribution of upscaling steps, the synthesis hotspots, and external developments until 2030. We also compared two predictions of TRL 9, one using data from TRL 5 and the other from TRL 6, to assess the influence of the pLCA prediction’s starting point. Finally, to better understand the influence of the activator on tire characteristics and tire environmental impacts, we conducted a pLCA on a tire made with ZnO@SiO_2_ including the expected change in rolling resistance.

## Methods

LCA is a methodology used by researchers, companies, and decision-makers to holistically assess the environmental impacts of a product or service throughout its lifetime. It is a well-established and widely used framework, extensively standardized through ISO14040 and 14044.^29,30^ In practice, it consists of an iterative process composed of four main steps: (1) the goal and scope definition, which sets the aim and limitations of the study, (2) the life cycle inventory (LCI) construction listing all unit flows and processes needed throughout the system’s life cycle, (3) the life cycle impact assessment where the LCI is converted into impacts in different categories, and (4) interpretation of the results.

### Goal and Scope

The goal of the LCA was to determine the environmental impact of producing the vulcanization activator ZnO@SiO_2_ to help guide its sustainable development. Therefore, three production scales were modeled, of which two were based on experimental data (TRL 5 and TRL 6), while the industrial scale (TRL 9) was predicted using pLCA for the year 2030. We modeled the industrial scale twice as TRLs 9a and 9b starting from TRL 5 and 6, respectively, and compared the predicted results. The framework of van der Hulst et al.^25^ was used as the methodological foundation of the pLCA.

**Figure 1:**
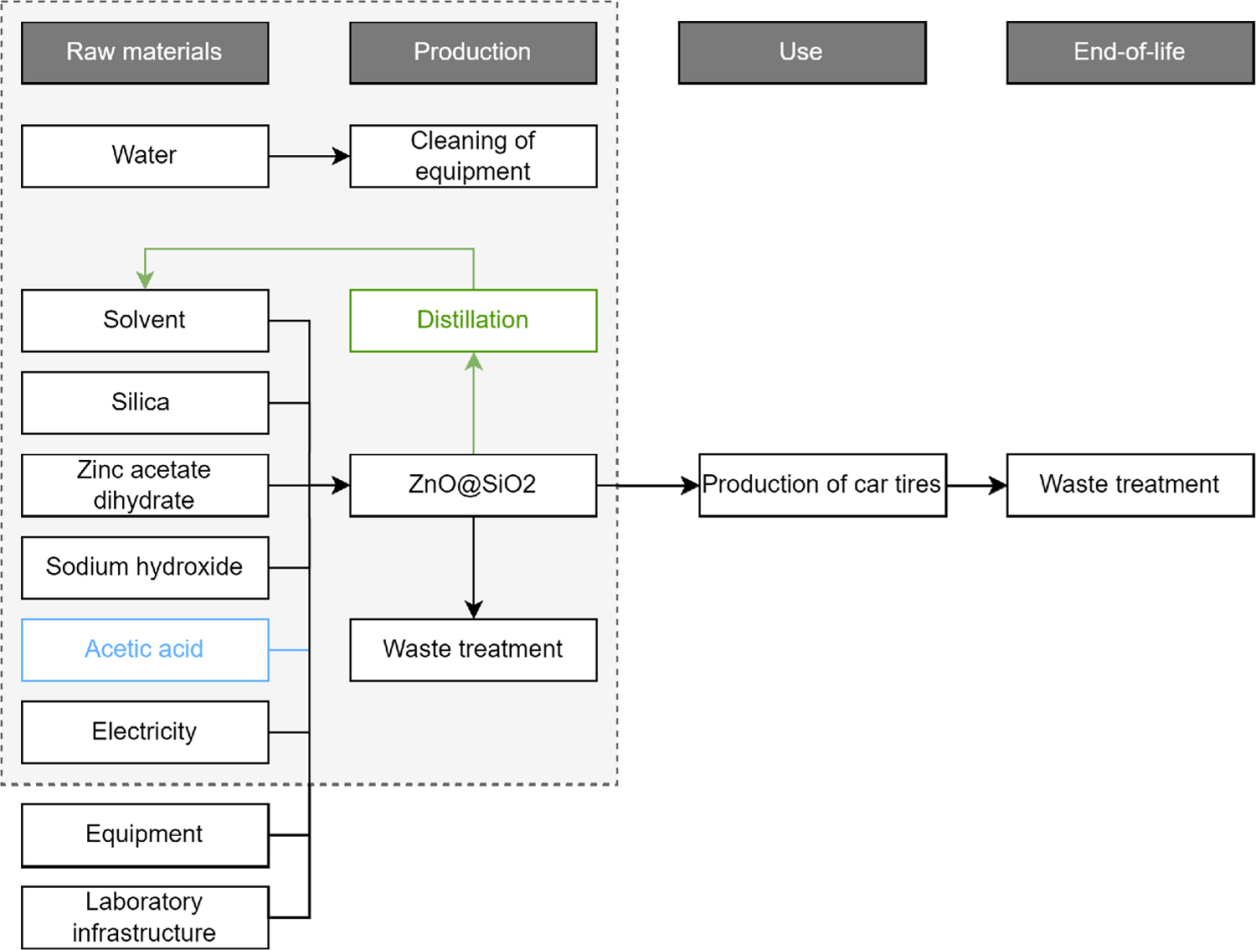
System boundaries of the LCA for the production of ZnO@SiO_2_. The system boundaries are depicted by the light gray, dotted outlined box. The acetic acid (in blue) is only used in TRLs 6 and 9b, while solvent recovery through distillation (in green) is only included in TRLs 9a and 9b.

The functional unit was producing 1 kg of ZnO@SiO_2_. Cradle-to-gate system boundaries were used, as illustrated in Figure 1, which include the provision of materials and production phases. The transport of the required chemicals from the factory to the experimental institute is also included. The production of the equipment is not included in the system boundaries because its contribution to the impact of this synthesis is assumed to be negligible. After production, ZnO@SiO_2_ is used as a curing activator in the production of car tires. The influence of the activator on the environmental impacts of a tire was estimated in a separate analysis (section 2.4).

### Life Cycle Inventory

Three main steps from the framework by van der Hulst et al.^25^ were followed, namely, TRL identification, process scaling, and modeling of developments at industrial scale. The European Horizon 2020 framework was used to identify the TRL of each of the three scales modeled.^31^ Process scaling was subdivided into size scaling, process changes, and process synergies, as defined by van der Hulst et al.^25^ Modeled developments at the industrial scale included external developments but excluded industrial learning for lack of required historical data. External developments in the energy, transport, and fuel sectors were modeled for 2030 following the “middle-of-the-road” shared socioeconomic pathway (SSP2) under a representative concentration pathway (RCP) of 2.6 W/m^2^.^32^ SSPs are narratives used to derive a set of future parameters (e.g., population, urbanization) that describe global socioeconomic changes until 2100.^33^ RCPs are narratives for how atmospheric greenhouse gas concentrations might develop.^34^ SSP2-RCP2.6 in particular is based on extrapolations of historic socioeconomic trends, while implementing constraints on greenhouse gas emissions such that the increase in global mean surface temperature is limited to 1.6–1.8 °C by 2100,^35^ in line with the Paris Agreement objective.^36^

TRLs 5 and 6 were based on experimental data, which were collected directly from the researchers conducting the upscaling.^12,13,37^ Data gaps were identified and filled by combining literature data with assumptions, which are listed in the Supporting Information (SI). The ecoinvent 3.9.1 inventory database was used to collect background data, using the cutoff version.^38^ It was assumed that all chemical waste was incinerated as hazardous waste. A generic ecoinvent entry for the treatment of spent solvent mixture was used.^38^ The energy use was modeled by using a European electricity mix for all TRLs to ensure comparability across scales.

For TRL 9, the upscaling framework of Piccinno et al.^28^ was used to predict industrial-scale inventory data. This approach brings together the main engineering-based calculations needed to quantify the energy usage of heated liquid-phase batch reactions and uses experimental data as a starting point. The industrial scale was modeled twice, using data from TRLs 5 and 6, resulting in TRLs 9a and 9b, respectively. This was used to assess the influence of the starting point on the upscaling framework.

**Figure 2:**
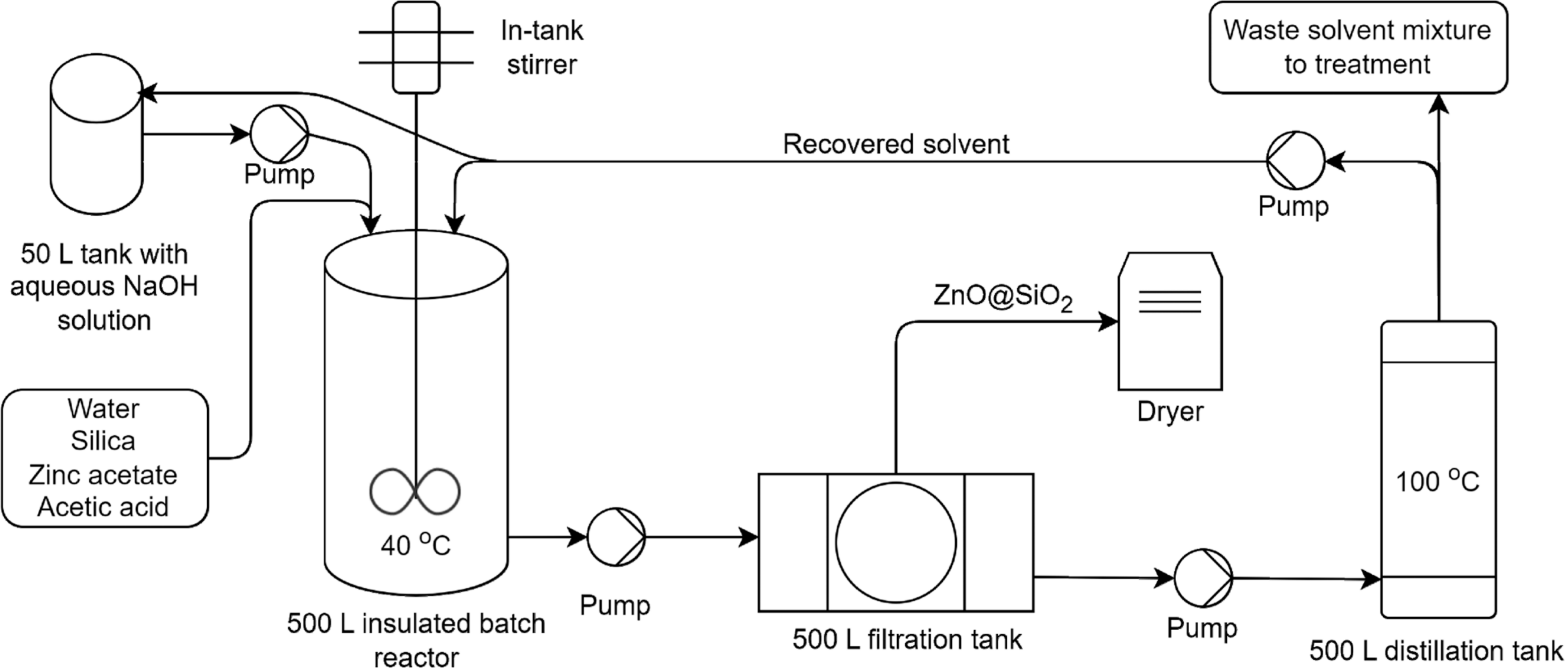
Flowchart of the production process of ZnO@SiO_2_ on industrial scale.

The synthesis procedure was upscaled to TRL 9 to fit industrial practices, as shown in the flowchart in Figure 2. The laboratory heating bath was assumed to be replaced with a heated liquid batch reaction in an insulated batch reactor with an in-tank stirrer. Distillation as process synergy was added to the industrial production process, where 68% of the solvent was assumed to be recovered, with the remaining 32% being treated as waste. Moreover, pumps were used to transfer the liquids automatically. In the SI, a detailed description is reported per process. The inventory data are summarized in Table 1 for TRLs 5, 6, and 9.

**Table 1:** Life Cycle Inventories (LCIs) for Each of the Production Scales Studied Per Functional Unit (i.e., Per kg of Activator Produced)

item	TRL 5	TRL 6	TRL 9a	TRL 9b	unit
Materials					
SiO_2_	9.27 × 10^–1^	8.89 × 10^–1^	9.27 × 10^–1^	8.89 × 10^–1^	kg/FU
NaOH	1.14 × 10^–1^	1.37 × 10^–1^	1.14 × 10^–1^	1.37 × 10^–1^	kg/FU
zinc acetate dihydrate	3.12 × 10^–1^	3.00 × 10^–1^	3.12 × 10^–1^	3.00 × 10^–1^	kg/FU
acetic acid		1.98 × 10^–3^		1.98 × 10^–3^	kg/FU
H_2_O, solvent	5.98 × 10^1^	2.00 × 10^1^	4.78 × 10^1^	1.60 × 10^1^	kg/FU
filter paper	8.73 × 10^–2^	3.05 × 10^–2^			kg/FU
transport	8.70 × 10^–1^	8.42 × 10^–1^	8.70 × 10^–1^	8.42 × 10^–1^	tkm/FU
Production					
energy use	3.71 × 10^2^	6.51 × 10^1^	4.09 × 10^1^	1.34 × 10^1^	kWh/FU
*heating*	1.25 × 10^2^	1.47 × 10^1^	1.17	4.26 × 10^–1^	kWh/FU
*stirring*	3.62	2.22	3.53 × 10^–4^	3.72 × 10^–4^	kWh/FU
*filtering*	1.79		1.00 × 10^–2^	1.00 × 10^–2^	kWh/FU
*drying*	2.37 × 10^2^	4.81 × 10^1^	4.42	4.42	kWh/FU
*fume hood*	3.10				kWh/FU
*pumping*	2.36 × 10^–1^	2.42 × 10^–2^	2.09 × 10^–3^	5.91 × 10^–4^	kWh/FU
*distillation*			3.53 × 10^1^	8.53	kWh/FU
H_2_O, used	7.14 × 10^1^	4.56	6.41 × 10^1^	2.61 × 10^1^	kg/FU
H_2_O, recovered			–2.85 × 10^1^	–6.89	kg/FU
Waste					
waste	6.01 × 10^1^	2.03 × 10^1^	1.34 × 10^1^	3.24	kg/FU

The starting point for external developments was the best-case scenario for TRLs 9a and 9b. Software package premise 1.8.1 was used to generate a background database for 2030 under the SSP2-RCP2.6 scenario.^39^ The ecoinvent 3.9.1 cutoff LCI database was used in premise as the background database.^38^ The year 2030 was chosen as this is a likely time frame for the activator deployment on the market. IMAGE was chosen as the underlying integrated assessment model for the SSP2-RCP2.6 scenario.^40^ Activity browser 2.9.4 was used to perform the LCA.^41^

### Impact Assessment and Interpretation

The ReCiPe 2016 method was used to perform the life cycle impact assessment, which can be used to calculate midpoint and end point indicators.^42^ Midpoint indicators represent environmental impacts at a specific point in the cause-effect chain, such as climate change or land use, while end point indicators represent environmental impacts at a higher level of aggregation, i.e., human health, biodiversity, and resource scarcity. ReCiPe includes three different perspectives representing groupings of assumptions and value choices. The individualist perspective is based on short-term interests and a best-case scenario for human technological adaptation, the hierarchist is a consensus-based middle point, and the egalitarian includes all known impact pathways and focuses on the long-term.^42^ All three perspectives were considered here as well as both midpoint and end point impacts. To match the needs of premise, notably for the future deployment of carbon capture technologies, some modifications were made to the characterization factors of ReCiPe for climate change impacts as listed in Table S5 in the SI.^43^

For visualizations, the upscaling of the chemical synthesis was broken down into steps following the framework of van der Hulst et al.^25^ The effect of size scaling and process changes were isolated from those of process synergies by modeling TRL 9 without synergies. Distillation is the only process synergy that we considered. We also modeled an alternative way to recover the solvent, namely, direct solvent reuse. Reuse of the water solvent was validated experimentally for one reuse cycle. Reuse up to four times is assumed to be practically achievable before the quality of the synthesis is affected, based on expert judgment for this reaction. The impacts of water use and waste stream were allocated equally between each synthesis that was part of a reuse cycle. An additional use of acetic acid was included for each cycle, which is used to correct the pH of the solvent before each reuse (approximately 0.3 g/kg of solvent).

### Potential Implications of the New Activator for the Life Cycle of the Tire

A tire made with the new activator is expected to have a lower rolling resistance, leading to an improved fuel consumption. To validate this hypothesis, scale-up tests were conducted at the University of Milano-Bicocca and at the tire manufacturer Pirelli Tire where rubber nanocomposites were prepared. Oscillatory dynamic-mechanical tests were performed on the rubber samples as well as on a control. The dissipative factor, also known as the loss factor or tan delta, was measured. The dissipative factor is recognized as a predictor for rolling resistance under the appropriate testing conditions.^44−47^ Based on those test results, as a first approximation, we assumed that the rolling resistance and lifetime of a tire made with the new activator would decrease by 14% compared to those of a reference commercial tire. The relative change in rolling resistance was assumed to lead to an equal relative change in lifetime due to the interconnected nature of tire characteristics, often summarized as the tire’s “magic triangle”.^48^ Alongside this most likely scenario, worst- and best-case funnel scenarios were modeled with decreases in rolling resistance and lifetime of 1 and 27%, respectively.

To estimate how the new activator influences the impacts of the full tire, we modeled the environmental impact of using it for tire production. We compared a commercial tire and a tire produced with the new activator by using the methodology developed in previous work.^8^ The tires modeled are average European passenger car tires. The system boundaries were extended to the life cycle of a tire from cradle to grave. We used a reference of driving 100,000 km using a passenger car tire. To link changes in tire characteristics to changes in tire environmental impacts, we used the relationships developed in our previous work.^8^ Results for the rubber mechanical tests and details on the methodology and related assumptions are reported in the SI.

## Results

### Activator Synthesis

**Figure 3:**
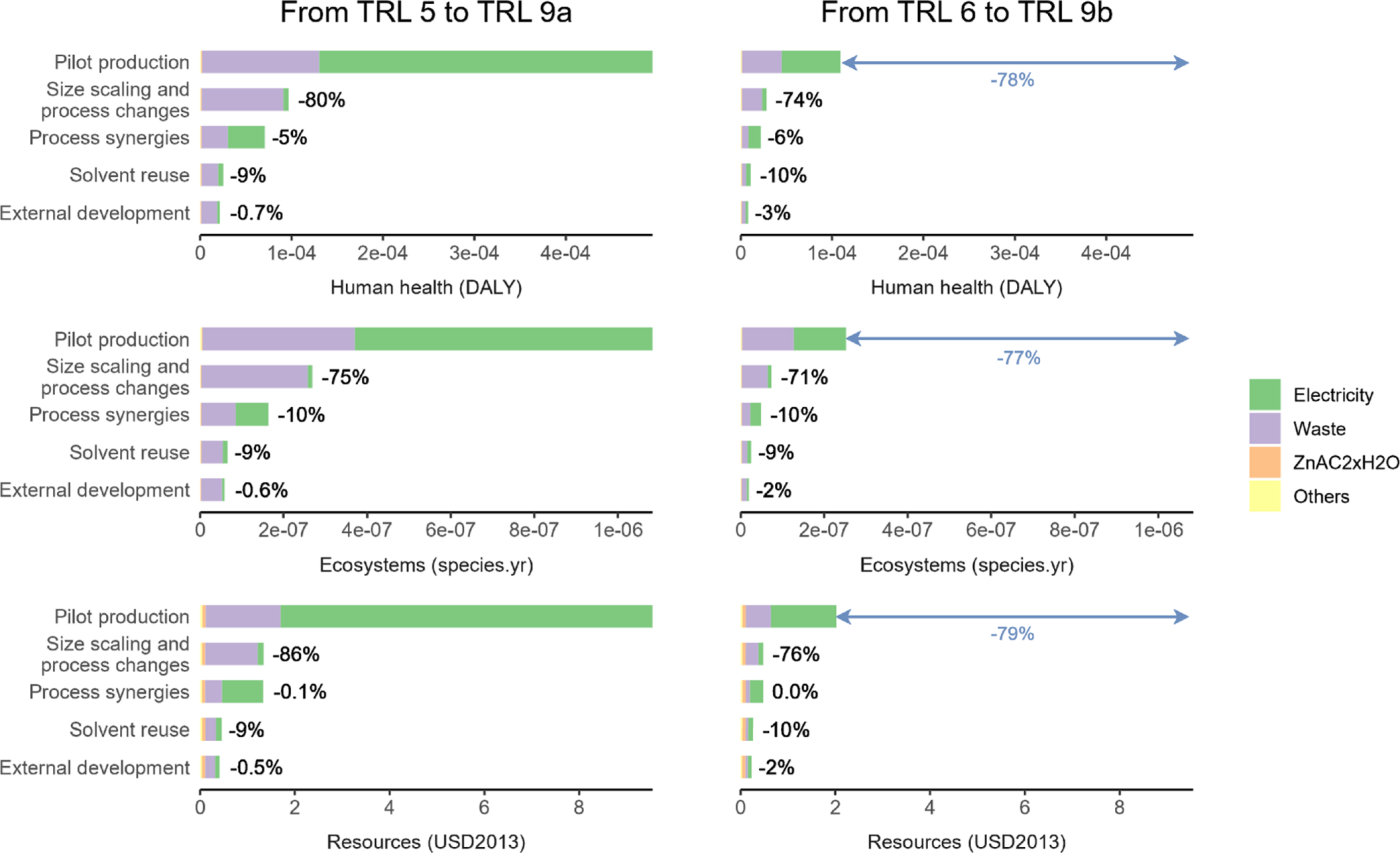
End point impacts of TRLs 5, 6, 9a, and 9b, per kilogram of activator synthesized in the hierarchist perspective. Hotspots that contribute less than 10% to the end point impact were grouped under “others”. Pilot production was based on experimental data, and subsequent steps to industrial scale were predicted using pLCA for size scaling, process changes, process synergies, and solvent reuse. External developments were forecasted based on the solvent reuse scenario, for 2030 under the IPCC SSP2-RCP2.6 scenario. Data labels show the change in impact relative to pilot production, while the double-sided arrows show the relative difference between TRL 5 and 6. Numerical values for the figure can be found in the SI (Table S6).

The end point results for all TRLs are shown in Figure 3 for the hierarchist ReCiPe perspective. A general trend of decreased impacts with an increased TRL is observed. The impact of distillation (process synergies in Figure 3) on resources is the only exception to that trend. This was caused by the heat requirement of distillation, which, unlike in other end points, was not counterbalanced by the benefits of having to treat less waste. Moreover, TRL 9b was predicted to cause the smallest impact for all end points.

Figure 3 also shows that a different starting point for the industrial prediction led to different TRL 9 impact predictions. TRL 9b with external developments had predicted impacts lower than those of TRL 9a by 63% for human health, 66% for ecosystems, and 44% for resources. This was due to the lower amount of solvent predicted to be used in TRL 9b, which led, in turn, to a lower amount of waste to be treated and of energy expended for heating and distillation. The same ranking of TRLs was found for the individualist and egalitarian ReCiPe perspectives (see Figures S3 and S4 in the SI).

Finally, it can be seen in Figure 3 that the two main environmental hotspots are waste treatment and electricity use. Waste treatment contributed to 26, 40, and 53% of the impact of TRLs 5, 6, and 9b (with external developments) on human health, respectively. Electricity use contributed to 74, 59, and 31% of the impact of TRLs 5, 6, and 9b (with external developments) on human health, respectively. The contribution of electricity use is affected by external developments, with impacts on human health decreasing from 44 to 31% when accounting for these external developments. Drying (48, 45%) and heating (25, 4%) were particularly impactful on human health for TRLs 5 and 6. For TRL 9b, distillation resulted in the highest impact on human health (40%), due to the high energy required to vaporize the solvent. The hotspots for ecosystems and resources followed a similar trend as those described for human health. Zinc acetate also contributed to 29% of resources for TRL 9b (with external developments), which emerged due to the lower contribution of other components. The same hotspots were found for the individualist and egalitarian ReCiPe perspectives (Figures S3 and S4 in the SI).

### Full Life Cycle Perspective of Activator

**Figure 4:**
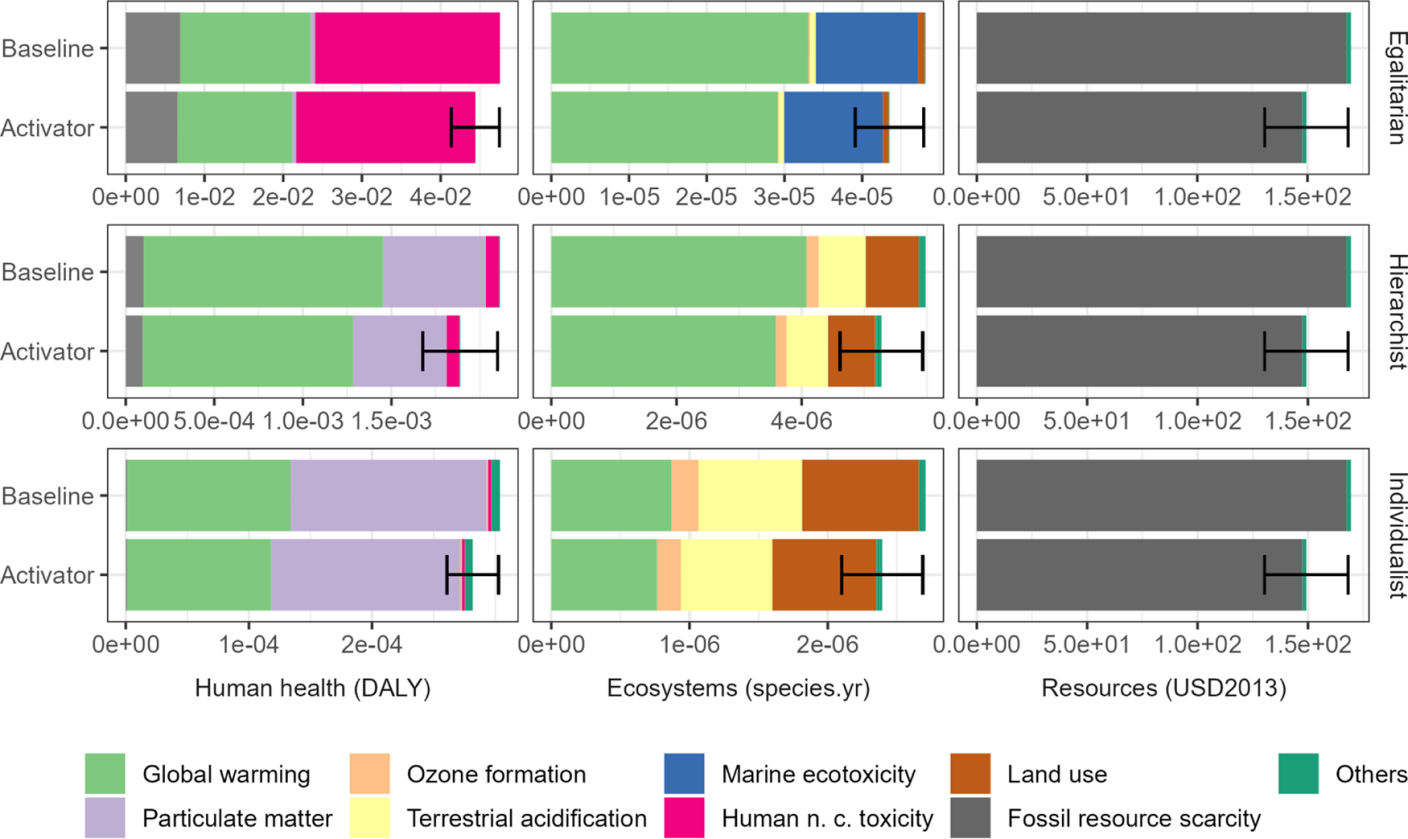
End point impacts of an average passenger car tire (“baseline”) and a tire made with the new activator (“activator”) per 100,000 km driven for 2030 (IPCC SSP2-RCP2.6). The “activator” scenario includes the expected decrease in rolling resistance (Potential Implications of the New Activator for the Life Cycle of the Tire section). The three ReCiPe perspective are shown as well as the contribution of midpoint categories to end point impacts. Midpoint categories that contribute less than 5% to end point impacts were grouped under “others”. The error bars show the results for the best- and worst-case scenario for the tire made with the new activator. “n.c.” stands for noncarcinogenic. Numerical values for the figure can be found in the SI (Table S7).

Figure 4 shows end point results for the comparison between a commercial tire and a tire produced with the new activator for the year 2030. The new vulcanization process was predicted to increase the impact of the tire production. However, when contextualized in the life cycle of the tire, this increase is negligible (<0.5%). Figure 4 shows that the improvement in rolling resistance counterbalances the higher impact of vulcanization and the decrease in tire lifetime, even in the worst-case scenario (i.e., the higher error bars in Figure 4). In the most likely scenario, significant improvements are expected when using the new activator, with decreases of 9 to 12% in all categories, except for human health in the egalitarian and individualist perspective where the decrease is 6%.

Figure 4 also shows that 8 midpoint categories contributed more than 5% to end point impacts. Global warming, particulate matter, human noncarcinogenic toxicity, and fossil resource scarcity were the main contributors to end point damage. Fuel production and combustion (well-to-wheel) are the main causes of global warming, particulate matter, and fossil resource scarcity impacts. Zinc release during the tire use phase, through tire wear particle emissions, was the main cause of the impacts on human noncarcinogenic toxicity and marine ecotoxicity.

## Discussion

Our results showed an expected reduction in the impact of the synthesis with an increase in TRL, which is in line with other studies. Piccinno et al.^19^ applied their upscaling framework in a case study of nanocellulose production and showed an 85% reduction of end point impacts from lab to industrial scale. Several other studies for the production of nanomaterials, aerogels, and solar panels have also found a reduction of at least 80%.^15,17,25^ Additionally, electricity consumption was shown to be the main contributing process to the overall impact during upscaling of chemical processes, with a lower relative contribution at higher TRLs.^18−20,49,50^ A high contribution of solvents and reactants was observed as well,^18,19^ which can be mitigated with solvent recycling.^51^

As mentioned by Thonemann et al.,^24^ one challenge that comes with pLCA is the uncertainty in data associated with predicting the future industrial scale’s LCI. The experimental data used here to compile the LCI for TRLs 5 and 6 were based on the best-case scenario estimation of the researchers from Fraunhofer ISC, as several synthesis routes were still being investigated. The proposed synthesis routes differed in the reaction temperature, filtration time, and dropping rate of the aqueous NaOH solution into the reaction mixture. These differences can have a large effect on the overall result since electricity use is one of the main hotspots.

TRL definition, the first step of a pLCA,^24,25^ was found to be a challenge as well. The EU Horizon 2020 framework^31^ leaves room for a researcher’s interpretation and subjectivity.^52^ Most noticeably, in this study, TRL 9a could be interpreted as a prediction of TRL 7 or 8. If that were the case, the comparison of TRL 9a against 9b would be in line with the observed trend of decreased environmental impacts with increased TRL. While this cannot be fully resolved without industrial-scale experimental data, the ambiguity in TRL definition should still be addressed by LCA practitioners conducting pLCAs.^52,53^ This can be done with more reliable characterizations which can be achieved by using TRL scales tailor-made for specific fields that are supported by quantitative indicators.^52^

The Piccinno upscaling framework was straightforward to use. However, there are limited choices for calculating the energy consumption of the filtration and drying technologies. Since energy consumption is a large contributor to the environmental impact, accurate predictions of the energy use of the specific technology expected to be used at the industrial scale are important. The framework should also include modeling solutions for waste treatment and estimations of the amount of liquid needed to wash the filtrate cake. Moreover, a relative solvent reduction higher than the 20% recommended by Piccinno et al.^28^ should be considered, particularly since this 20% is recommended disregarding the starting point of the prediction. Indeed, a rate of 67% was empirically observed here between TRLs of 5 and 6. This parameter was shown to have a high influence on the results, with reductions of impact of about 59% across end point categories (see Figure S2 in the SI). This finding shows that LCA practitioners should carefully consider the starting point of their pLCA predictions.

For scaling up the production process of ZnO@SiO_2_, our recommendations are to limit energy use and to investigate low-environmental impact waste treatment options. The impacts of energy and waste can be mitigated by minimizing the amount of solvent used, which can be achieved with distillation or direct reuse. It was shown that a decrease in solvent use resulted in an approximately linear decrease in impacts (Figure S2 in the SI). Future research should also look into the effects of industrial learning.

Regarding the effects of the new activator on tires, the main assumption is the link between the mechanical tests on rubber composite and the observed changes in tire characteristics. Due to the uncertainty linked with those assumptions, we could provide only a preliminary estimate of the environmental benefits that can be achieved by using the new activator. The worst-case scenarios of tire characteristics changes demonstrated that a small improvement in rolling resistance still outweighs the increase in impacts due to both the synthesis of the new activator and the decrease in tire lifetime.

Future research on the properties and environmental impacts of ZnO@SiO_2_ should aim to conduct tests on full-size tires, specifically measuring the rolling resistance. Furthermore, the research into the new activator was motivated by the thought to decrease the amount of zinc in the tire, and thus decreasing the amount of zinc released into the environment. However, this could not be assessed yet. Decreasing the amount of zinc in the tire, would lead to lower impacts, especially related to impacts on human noncarcinogenic toxicity and marine ecotoxicity. Moreover, it should be noted that the results presented here are only valid for a passenger car tire used with a petrol internal combustion engine. The conclusions may be affected if a diesel or electric car were modeled instead, or for another type of vehicle.

## Conclusions

This study aimed to assess the environmental impacts of synthesizing and using ZnO@SiO_2_, a new tire vulcanization activator. The industrial scale impacts of the synthesis were predicted using pLCA. We found that the end point impacts of the synthesis decreased by 89 to 96% between the pilot and industrial scales. The largest contributors to the synthesis of ZnO@SiO_2_ were the electricity consumption and the waste treatment of the solvent. Using this activator for tire production led to predicted potential benefits over the tire life cycle of 6 to 12% across end point categories when compared to a commercial tire in the year 2030. Those gains were due to the expected decrease in rolling resistance, which outweighed the additional environmental costs of the synthesis as well as the potential decrease in lifetime. Future research may focus on evaluating the influence of industrial learning as well as on further quantifying the decrease in zinc content and decrease in rolling resistance expected for a tire made with the new activator.

## Supporting Information Available

Additional methodological information can be found about the synthesis routes (section 1.1); LCI of zinc acetate (1.2); prediction of the industrial LCI (1.3); rubber mechanical tests (1.4); LCI of a tire made with the new activator (1.5); and changes to ReCiPe (1.6); a list of the main assumptions related to the LCI (section 2); collection of results underlying the figures presented here (section 3.1), including TRL comparison for the individualist and egalitarian ReCiPe perspectives; and the influence of the relative solvent reduction parameter (section 3.2) (PDF)
