# Supplementary material for: Environmental Impact Prediction of a New Tire Vulcanization Activator
Source: ACS Sustain Chem Eng. 2024 Apr 10;12(16):6102–10. doi: 10.1021/acssuschemeng.3c06640 (PMC11041116; doi:10.1021/acssuschemeng.3c06640)
Supplement: Supplementary file 1 — sc3c06640_si_001.pdf [file sc3c06640_si_001.pdf]

Supporting information

AUTHOR NAMES

Thomas Hennequin [a], Lotte van Vlimmeren [a], Silvia Mostoni [b], Francesca Rita Pomilla [b], Roberto Scotti [b,c], Claudia Stauch [d], Mitchell K. van der Hulst [a,e], Mark A.J. Huijbregts [a], Rosalie van Zelm,[a].*

AUTHOR ADDRESS

[a] Department of Environmental Science, Radboud Institute for Biological and Environmental Sciences, Radboud University, Nijmegen, The Netherlands.

[b] Department of Material Science, INSTM, University of Milano-Bicocca, Via Roberto Cozzi 55, 20125 - Milano, Italy.

[c] Institute for Photonics and Nanotechnologies-CNR, Via alla Cascata 56/C, 38123 Povo (TN), Italy

[d] Fraunhofer Institute for Silicate Research, Würzburg, Germany.

[e] Expertise Group Circularity & Sustainability Impact, TNO, P.O. Box 80015, 3508 TA, Utrecht, The Netherlands

*Corresponding Author: Rosalie van Zelm, rosalie.vanzelm@ru.nl.

1. Method

1.1. Synthesis routes

ZnO-NP@SiO_2-NP was first produced by researchers from the University of Milan-Bicocca (Susanna et al., 2015, 2017) at TRL3, small lab scale. The synthesis was later upscaled by researchers of the Fraunhofer ISC (Bittel, 2021; Fett, 2019; Kevin, 2020) with production steps at TRLs 4, 5, and 6. During upscaling, several process changes were made. The main differences between the TRLs are summarized in Table S1. The most important process change was the replacement of absolute ethanol as solvent by deionized water. Using absolute ethanol as solvent was not possible in TRL 4 and higher due to financial and safety concerns (Fraunhofer ISC, 2020). Therefore, ethanol and water were studied at TRL 4. Since empirical results showed that water is financially and technically ideal for the upscaling process (Fraunhofer ISC, 2020), water was chosen as solvent for TRLs 5 and up.

The different properties of the solvents affect the chemical reaction, resulting in a change in the synthesis route and thus in the experimental procedure. One of these changes was the increase in reaction temperature from 65˚C to 80˚C for the first water synthesis route (TRL 4). The reaction temperature decreased to 40˚C for TRL 5, because of experimental optimization. The researchers at Fraunhofer ISC have also chosen to use oven drying for TRL 4-6 instead of drying at room temperature (RT). The difference in yield between TRL 4a, the scenario with ethanol as solvent, and TRL 4b, with water as solvent, can be explained by a lowered experimental efficiency. The synthesis route was already optimized for ethanol, but not yet for water. The difference in yield between TRL 3, 4, and 5, however, cannot be explained by process changes only, but mainly by size scaling. Another notable change of process is the transition from lab equipment in TRL5 to an industrially sized batch reactor in TRL6.

Table S1. An overview of the differences between the synthesis routes for TRL 3, 4a, 4b, 5 and 6.

	**TRL 3**	**TRL 4a**	**TRL 4b**	**TRL 5**	**TRL 6**
**Scale**	Proof of concept	Validation in laboratory	Validation in laboratory	Validation in relevant environment	Demonstration in relevant environment

**Solvent**	Absolute ethanol	Ethanol	Deionized water	Deionized water	Deionized water
**Reaction temp.**	65˚C	65˚C	80˚C	40˚C	40˚C
**Drying method**	Room temp.	Oven drying	Oven drying	Oven drying	Oven drying
**Equipment**	Laboratory	Laboratory	Laboratory	Laboratory	Batch reactor
**Yield**	62%	82%	68%	96%	100%
**Product**	1.87 g	4.99 g	4.13 g	59.32 g	1.69 kg

For all TRLs, the reactants used in the synthesis routes remained the same: silica (SiO_2), zinc acetate dihydrate ($ZnAc_2{\cdot}H_2O$), and sodium hydroxide (NaOH). TRL 6 is the only exception where acetic acid was also used in order to reach a higher yield. In TRL 3, ZnO-NP@SiO_2-NP is produced using a sol-gel procedure (Susanna et al., 2015). The consecutive steps are (1) dispersion of SiO_2 in ethanol using a sonicator for 10 min, (2) adding $ZnAc_2{\cdot}H_2O$ and NaOH under stirring while heating, (3) filtering the nanoparticles, (4) washing them four times with ethanol, and (5) drying at room temperature (Susanna et al., 2017).

The steps for TRL 4a are (1) heating NaOH in ethanol for 55 min, (2) addition of $ZnAc_2{\cdot}H_2O$ and later SiO_2 under stirring, (3) filtering using a vacuum pump, (4) washing the nanoparticles 3 times with water, and (5) drying them in an oven overnight. The main differences in TRL 4b are the increased reaction temperature, the increased heating time from 55 to 75 min, and the increased drying time. For TRL 5, the procedure is changed to (1) heating the distilled water, (2) adding SiO_2 and $ZnAc_2{\cdot}H_2O$ simultaneously while stirring, (3) dropping a NaOH solution using a peristaltic pump, (4) filtering of the nanoparticles by vacuum pumping, and (5) drying in an oven at 107 ˚C. Finally, in TRL 6 the procedure was (1) dissolution of NaOH in the water solvent using an ultrasonic bath, (2) filling, mixing, and heating of batch reactor to 40 ˚C, (3) addition of NaOH/water solution with pump followed by heated and stirred reaction time, (4)

emptying of reactor content and filtering with sieves and vacuum pump, and (5) drying in an oven at 60 °C.

1.2. Modelling zinc acetate

The production of $ZnAc_2 \cdot H_2O$ was modeled according to Fridrihsone et al. (2020) using 37% Zinc Oxide, 55% Acetic Acid, 8% water by weight. All individual components were modeled using ecoinvent entries (Wernet et al., 2016). The energy required for the synthesis was based on the enthalpy of formation, which was calculated by Hughes and Navrotsky (2011) to be 66.02 kJ/mol on lab scale. Since production at the industrial scale is more energy-intensive than the theoretical energy use due to heat and energy losses, the lab scale energy should be multiplied by 4.2 to account for the losses (Mehrkesh & Karunanithi, 2013).

1.3. Predicting the industrial LCI

1.3.1. Industrial prediction from TRL 5 (TRL 9a)

Each of the production processes is scaled up individually before linking them. Table S2 summarizes the scale-up including the equations used for calculating the energy use of industrial processes. The parameters and notations used are the same as in Piccinno et al (2016). For each process, the upscaling calculations are described below. The assumptions made can be found in section 2.

Reactants – The starting point of the framework is to determine the amount used for reactants at TRL 9a. To ensure that the reactants are used in the same relative quantity, the amounts of SiO_2, NaOH, and $ZnAc_2 \cdot H_2O$ were scaled linearly from TRL 5 to a production with a yield of 10 kg ZnO-NP@SiO_2-NP.

Solvent – On a laboratory scale, solvents are used inefficiently due to the small quantities of the reactants. Since there was no exact concentration or ratio required, the amount of distilled water could be scaled with a 20% relative reduction compared to TRL 5.

Reactor size – The mass of the reaction mixture was estimated to be approx. 490 kg. Because of this, a reactor size volume of 500 L was assumed. An additional tank is needed for the aqueous NaOH with a reaction mixture mass of 30.25 kg.

Heating energy – The reaction temperature is 40°C and the energy required to maintain this temperature during the reaction was calculated assuming a cylindrical reactor, with $C_p = 4181$ J.kg^{-1}.K^{-1}; $m_{mix} = 491.7$ kg; $T_r = 313.15$ K, $t = 124$ min, $A = 3.716$ m^2, $ka/s = 0.56$ WK^{-1}, $\eta_{heat} = 74\%$.

Stirring energy – The stirring energy was calculated assuming an axial flow impeller, with $N_p = 0.79$, $\rho_{mix} = 1028.32$ kg/m^3, $N = 3.83$ s^{-1}, $d = 0.296$ m, $t = 184$ min, $\eta_{stir} = 90\%$.

Filtration – According to the framework, the energy used for filtration ranges from 1 to 10 kWh per ton of dry material, with 1 kWh for larger particles and 10 kWh for small particles. Since the filtration technique fit for upscaling is still being studied, the upper limit of 10 kWh is chosen for the ZnO-NP@SiO_2-NP nanoparticles, with $m_{dry\_particles}$ = 10 kg. In addition to the equipment used for filtration, washing liquid is required to remove waste from the product. The amount of washing liquid is estimated to be three times the mass of the product pre-drying.

Drying – The framework does not distinguish the energy use for different drying techniques but presents an equation to calculate the energy use for vaporization of the solvent. This equation was used with $T_{boil} = 100$ °C, $\Delta H_{vap} = 2.26$ MJ.kg^{-1}, $m_{vap} = 6.193$ kg, $n_{dry} = 80\%$.

Water use for cleaning – The framework does not provide any upscaling calculations for cleaning. Therefore, the water use for cleaning was scaled linearly.

Waste treatment – Piccinno gives options for waste treatment but does not include it in the upscaling framework itself. Two options were studied, recovering the solvent distillation, thereby reducing the amount of waste and the amount of solvent needed, and treating all waste as hazardous waste. Without distillation, all filtrate was treated as hazardous waste as well as the water used to wash the filtration cake.

Table S2. Summary of the scale-up to TRL 9 including the equations and parameters used (Piccinno et al., 2016).

Laboratory scale process	Scale-up process	Scale-up calculation
Reaction under heating	Heated liquid batch reaction in an insulated batch reactor with an in-tank stirrer	$Q_{react} = \frac{C_p * m_{mix} * (T_r - T_0) + A * \frac{k_a}{s} * (T_r - T_0) * t}{\eta_{heat}}$
Mixing by magnetic stirrer	In-tank stirring	$E_{stir} = \frac{N_P * \rho_{mix} * N^3 * d^5 * t}{\eta_{stir}}$
Filtration by vacuum pump	Filtration	1-10 kWh per ton of dry material<br>Add washing liquid

Drying in oven	Drying	$Q_{dry} = \frac{C_{p,liq} * m_{liq} * (T_{boil} - T_0) + \Delta H_{vap} * m_{vap}}{\eta_{dry}}$
Waste incineration	Hazardous waste/ Distillation	$Q_{dist} = \frac{C_p * m_{filt} * (T_{boil} - T_0) + \Delta H_{vap} * m_{dist}}{\eta_{heat} - 0.1}$ Add cooling water for waste
Manual transferring solution	Pumping	$E_{pump} = 55 * m_{pumping}$

Step 3 of the framework is to link the individual processes to each other. The links studied here were the distillation and the transfer of the liquid.

Distillation – During distillation, all the filtrate is boiled, and part of the solvent can be recovered. According to Piccinno, the average recovery rate in industrial processes is 68%. For water, a recovery rate of 56-75% was found by Santos et al. (2020). The energy use for distillation was therefore calculated using the average 68% recovery rate, with m_{dist} = 285.4 kg. This yields 134 kg of waste that must be cooled using 0.027 kg of cooling water per kg of waste.

Pumping – The link between all process steps is to transfer the solution. On the laboratory scale, this is done manually, but on industrial scale pumps are used. Without distillation, three pumps are needed; to transfer the aqueous NaOH solution to the 500 L insulated batch reactor; to transfer the reaction mixture to the filtration equipment; to transfer the filtrate to the waste treatment equipment. When distillation is included, two additional pumps are needed; to transfer the filtrate to the distillation tank; to transfer the distilled solvent to the 500 L insulated reactor batch.

1.3.2. Industrial prediction from TRL 6 (TRL 9b)

The second upscaling from TRL 6 to TRL 9b was largely based upon the same methodology. For each process, the upscaling calculations are described below when it deviates from the upscaling from TRL 5. The upscaling was more straightforward as TRL 6 is closer to the industrial scale.

Reactants – The starting point of the framework is to determine the amount used for reactants at TRL 9b. To ensure that the reactants are used in the same relative quantity, the amounts of SiO_2, NaOH, acetic acid, and $ZnAc_2 \cdot H_2O$ were scaled linearly from TRL 6 to a production with a yield of 10 kg ZnO-NP@SiO_2-NP.

Homogenizing – While Piccinno does mention different types of homogenization, only the rotor-stator type is modeled. Given its relatively low contribution to the overall energy consumption (less than 0.1%) at TRL6, it was assumed to be negligible at TRL9b.

Heating energy – The reaction temperature is 40°C and the energy required to maintain this temperature during the reaction was calculated using the same approach as for TRL 9a, with C_p = 4181 $Jkg^{-1}K^{-1}$; m_{mix} = 173.2 kg; T_r = 313.15 K, t = 263 min, A = 3.716 m^2, ka/s = 0.56 WK^{-1}, η_{heat} = 74%.

Stirring energy – The stirring energy was calculated with an axial flow impeller, with N_p = 0.79, ρ_{mix} = 1082.82 kg/m^3, N = 0.82 s^{-1}, d = 0.296 m, t = 408.5 min, η_{stir} = 90%.

Distillation – As for TRL 9a, the energy use for distillation was calculated using the average 68% recovery rate, with m_{dist} = 68.9 kg. This yields 32.4 kg of waste.

1.4. Rubber mechanical tests

Scale-up tests were conducted at the University of Milano-Bicocca (UniMiB) where four rubber nanocomposites were prepared in a Haake mixer. A reference and three samples were tested, the characteristics of the sample are described in Table S3. An oscillatory dynamic-mechanical test was conducted on each sample as well as on the reference, both for isoprene and natural/butadiene rubber compounds.

Table S3. Characteristics of the rubber nanocomposites prepared and tested by UniMiB

Sample	Zn:OH	T (°C)	Time (min)
SV11	1:2.5	40	100
SV12	1:4	40	180
SV13	1:2.5	40	180

In a typical oscillatory dynamic-mechanical test, the application of an oscillatory shear strain γ of angular frequency ω, results in a sinusoidal stress σ, as expressed in the following equations:

$$\gamma(t) = \gamma_0 + \sin(\omega t) \quad S1$$

$$\sigma(t) = \sigma_0 + \sin(\omega t + \delta) = \sigma_0[\sin(\omega t)\cos(\delta) + \cos(\omega t)\sin(\delta)] \qquad S2$$

Where δ is the phase angle.

G* is a complex modulus determined by an in-phase elastic component (G') and an out-phase dissipative component (G''). The ratio between G'' and G', called tanδ (dissipative factor), expresses the energy lost in a cyclic deformation. Both G' and G'' depends on the frequency and on the temperature. They are defined by the following equations:

$$G' = \frac{\sigma_0 \cos(\delta)}{\gamma_0} \qquad G'' = \frac{\sigma_0 \sin(\delta)}{\gamma_0} \qquad S3$$

The trend of tanδ at low frequency is a predictor of the rolling resistance (Larry R. Evans et al., 2009; Masanori Kan et al., 1984; Okel, 1998; Vleugels et al., 2015). The results of the oscillatory dynamic-mechanical test can be found Figure S1 for tanδ. The test was repeated at the Pirelli tire manufacturer under similar conditions, corroborating UniMiB's results.

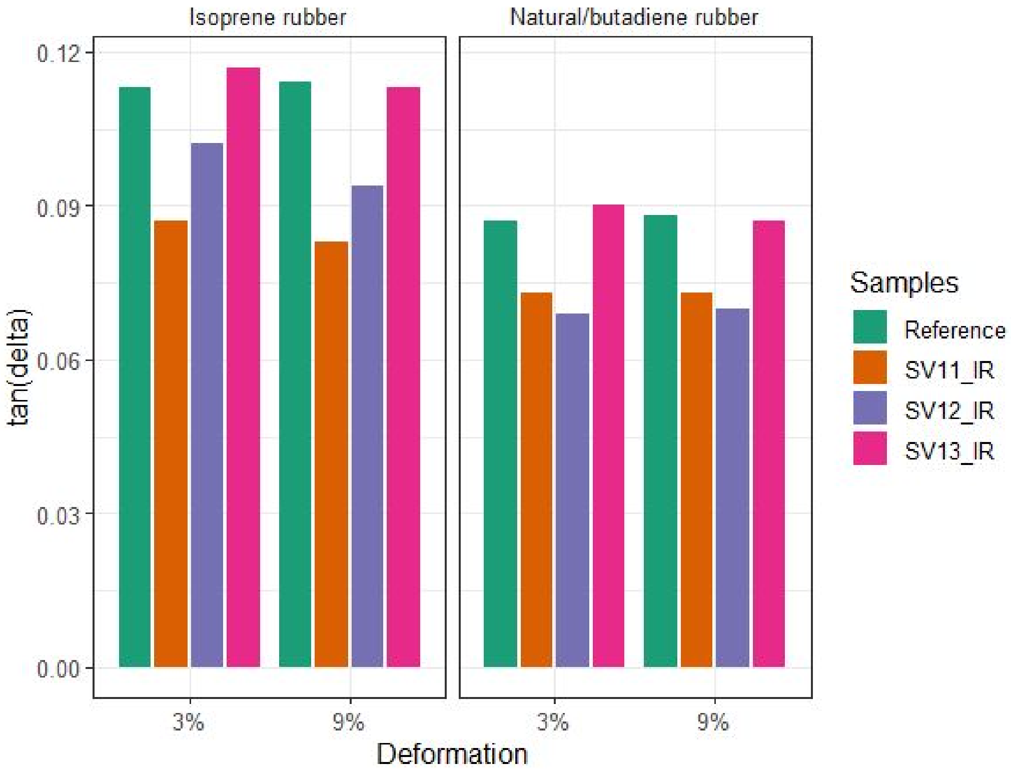

Figure S1. Dissipative factor (tanδ) measurements resulting from the UniMiB oscillatory dynamic-mechanical testing for natural and isoprene rubbers.

1.5. LCI for tire made with new activator

New LCI values for the tire vulcanization were calculated based on the experimental results presented in the previous section. For lack of a quantified relationship, we assumed that a change in the dissipative factor compared to the control would result in an equivalent relative change in rolling resistance. We used the results for a 9% strain and averaged the isoprene and natural rubbers results to calculate the relative change in dissipative factor.

Due to the interconnected nature of tire characteristics, often summarized as the tire's "magic triangle" (Tullo, 2009), we also assumed that a relative change in rolling resistance would lead to an equal relative change in lifetime. The uncertainty of those assumptions as well as that of the test measurements were accounted for with a funnel scenario approach where we modeled worst- and best-case scenarios alongside the most likely outcome. An overview of the changes in tire characteristics modeled for those scenarios is shown in Table S4. To link changes in tire

characteristics to changes in tire environmental impacts, we used the relationships developed in our previous work (Hennequin et al., 2022).

Table S4. Expected changes in tire characteristics due to the use of the new activator. Worst and best cases were modeled to account for the uncertainty of the mechanical tests and of their interpretation.

**Scenario**	**Rolling resistance**	**Lifetime**
Reference	0%	0%
Worst	-1%	-1%
Most likely	-14%	-14%
Best	-27%	-27%

1.6. Changes to ReCiPe

By using a prospective database created with premise, some modifications needed to be made to the characterization factors of climate change impact categories in ReCiPe. New technologies are added to ecoinvent by premise, which have environmental flows for which no characterization factors are included in the standard ReCiPe LCIA method. These relate to the removal of CO2 from the air and storing it in the soil, as well as fugitive emissions of hydrogen. Furthermore, characterization factors for non-fossil methane were adjusted to correspond with the characterization factor for fossil methane, minus 2.75 kg CO_2 eq., as proposed by Muñoz & Schmidt (2016). These changes are needed to cover the future deployment of hydrogen supply chains as well as carbon capture from the air or biogenic sources. Values advised by the developers of premise (Sacchi, 2023) were used and adapted to ReCiPe climate change characterization factors and perspectives as listed in Table S5. The values of Table S5 were converted to endpoint characterization factors using ReCiPe mid-to-endpoint factors for each perspective (Huijbregts et al., 2017).

Table S5. Changes to ReCiPe climate characterization factors made to match premise. I, H, and E stand for the three ReCiPe perspectives, namely, individualist, hierarchist, and egalitarian, respectively.

**Substance**	**Unit**	**I**	**H**	**E**
Hydrogen	kg CO_2 eq.	33	11	11

Fossil methane	kg CO_2 eq.	85	36	4.9
Biogenic methane	kg CO_2 eq.	82.25	33.25	2.15
Carbon dioxide, in air	kg CO_2 eq.	-1	-1	-1
Carbon dioxide, non-fossil, resource correction	kg CO_2 eq.	-1	-1	-1
Carbon dioxide, non-fossil	kg CO_2 eq.	1	1	1

2. List of assumptions

It was assumed that all waste is treated as a generic spent solvent mixture (i.e. incinerated as hazardous waste). This might be an oversimplification of the waste treatment resulting in an overestimation of its impact. Another scenario could be treating the waste as wastewater since it is an aqueous solution. However, this might not be possible due to the traces of zinc in the wastewater depending on local wastewater regulations.

Several additional assumptions were made regarding the compounds and equipment used. The assumptions listed below are general assumptions that are valid for all scales:

- Equipment production was assumed to be negligible because it is used for a larger number of reactions during its lifetime. The production of the equipment and the transport to the production site were excluded, only the energy use of the equipment was included.
- For the energy use, the amount of electricity consumption was calculated by multiplying the power of the equipment with the amount of time the equipment is used.
- Fume hoods have a power of 25W when only fluorescent light is turned on, but of 60W when the microprocessor is also in use (ESCO, 2022).
- The filter paper used in combination with the vacuum filter consists of cellulose fibers with a density of 88 g/m2 (Merck, 2023). The diameter of the filtration paper is 15cm when using ethanol and 12.5cm for water.

Moreover, some assumptions are TRL specific. These assumptions are listed below.

TRL 5:

- A standard laboratory vacuum pump with a power of 130W (Fisher Scientific, 2016) was modelled.

- For the transport, it was assumed that the production sites of Solvay in Belgium and Roth in Germany are located in Beveren (800km from München) and Karlsruhe (300km from München), respectively. It was assumed that the ethanol is also bought at Roth in Germany
- The oven used has a power of 1400W. It was assumed that the oven is electric and turns on and off during its use as needed. Based on the graphs by Landi et al. (2019) it was estimated that the oven requires five minutes to reach the desired temperature. Afterwards the oven was estimated to be on 50% of the time it is used.

TRL 9:

- For prediction of TRL 9, the Piccinno framework mentions that the industry mostly uses gas for the heating process. However, Safe-Vulca partners from the Fraunhofer ISC have supplied documentation on the reactor they used for TRL 6. This reactor is electric and is comparable to the size used for 10 kg product. Therefore, it was assumed that the industrial reactor can be heated using electricity.
- For transport, it is unknown where the reactants would be shipped from. Therefore, the same distance as TRL4/5 was used.
- Since no pre-drying mass was reported in TRL 6 to be used for the TRL 9b calculations, it was assumed that the ratio of solid content was the same as in TRL 5 (approx. 14%).

3. Results

3.1. Endpoint results for all TRL

Table S6 shows a collection of endpoint impacts for the TRLs considered and for the three ReCiPe perspectives (Huijbregts et al., 2017). Table S7 shows the endpoint results for the tire comparison and for the three ReCiPe perspectives.

Table S6. Endpoint impacts for TRLs 5, 6, 9a, and 9b, per kg of activator synthesized. The three ReCiPe perspectives are shown. The "Start" columns indicates the starting point of the industrial prediction. Figure 3 in the main body of the article shows these results for the hierarchist perspective.

Start	Upscaling step	Perspective	Human health (DALY)	Ecosystems (species.yr)	Resources (USD2013)
TRL 5	External development	Egalitarian	5.13E-04	5.84E-07	4.22E-01
TRL 5	External development	Hierarchist	2.15E-05	5.85E-08	4.10E-01
TRL 5	External development	Individualist	2.34E-06	1.68E-08	4.11E-01
TRL 5	Solvent reuse	Egalitarian	6.11E-04	6.54E-07	4.80E-01
TRL 5	Solvent reuse	Hierarchist	2.52E-05	6.52E-08	4.54E-01
TRL 5	Solvent reuse	Individualist	3.24E-06	2.04E-08	4.55E-01
TRL 5	Process synergies	Egalitarian	2.29E-03	1.90E-06	1.49E+00
TRL 5	Process synergies	Hierarchist	7.06E-05	1.64E-07	1.33E+00
TRL 5	Process synergies	Individualist	9.81E-06	6.33E-08	1.33E+00
TRL 5	Size scaling and process changes	Egalitarian	1.75E-03	2.43E-06	1.37E+00
TRL 5	Size scaling and process changes	Hierarchist	9.68E-05	2.69E-07	1.33E+00
TRL 5	Size scaling and process changes	Individualist	1.16E-05	7.26E-08	1.34E+00
TRL 5	Pilot production	Egalitarian	1.80E-02	1.36E-05	1.10E+01
TRL 5	Pilot production	Hierarchist	4.95E-04	1.08E-06	9.55E+00
TRL 5	Pilot production	Individualist	7.11E-05	4.66E-07	9.53E+00
TRL 6	External development	Egalitarian	2.78E-04	2.37E-07	2.41E-01
TRL 6	External development	Hierarchist	7.94E-06	1.96E-08	2.32E-01
TRL 6	External development	Individualist	9.07E-07	6.73E-09	2.32E-01

TRL 6	Solvent reuse	Egalitarian	3.58E-04	2.93E-07	2.86E-01
TRL 6	Solvent reuse	Hierarchist	1.08E-05	2.50E-08	2.64E-01
TRL 6	Solvent reuse	Individualist	1.53E-06	9.62E-09	2.64E-01
TRL 6	Process synergies	Egalitarian	7.66E-04	5.96E-07	5.31E-01
TRL 6	Process synergies	Hierarchist	2.18E-05	4.88E-08	4.75E-01
TRL 6	Process synergies	Individualist	3.12E-06	2.00E-08	4.75E-01
TRL 6	Size scaling and process changes	Egalitarian	6.34E-04	7.24E-07	5.00E-01
TRL 6	Size scaling and process changes	Hierarchist	2.81E-05	7.42E-08	4.76E-01
TRL 6	Size scaling and process changes	Individualist	3.55E-06	2.23E-08	4.78E-01
TRL 6	Pilot production	Egalitarian	3.56E-03	2.94E-06	2.28E+00
TRL 6	Pilot production	Hierarchist	1.09E-04	2.52E-07	2.02E+00
TRL 6	Pilot production	Individualist	1.52E-05	9.86E-08	2.02E+00

Figure 3 in the main body of this article was presented for the hierarchist perspective. Figures S3 and S4 here show the same results for the individualist and egalitarian perspectives, respectively. The rankings of TRLs and the main hotspots are the same in all three ReCiPe perspectives. A notable difference is that distillation (i.e. process synergy) is not favorable for human health impacts in the egalitarian perspective.

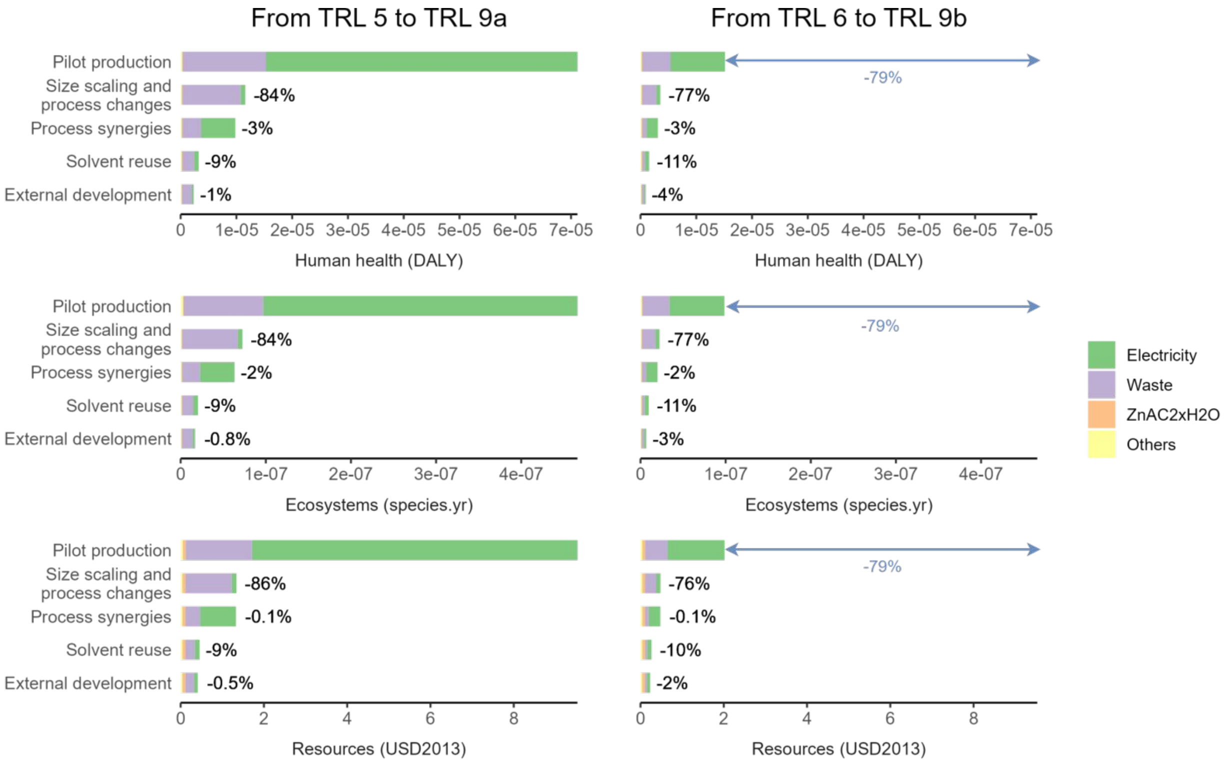

Figure S2. Endpoint impacts of TRLs 5, 6, 9a, and 9b, per kg of activator synthesized in the individualist perspective. Hotspots that contribute less than 10% to the endpoint impact were grouped under "Others". Pilot production was based on experimental data, and subsequent steps to industrial scale were predicted using pLCA for size scaling, process changes, process synergies, and solvent reuse. External developments were forecasted based on the solvent reuse scenario and for 2030 under IPCC SSP2-RCP2.6. Data labels show the change in impact relative to pilot production while the double-sided arrows show the relative difference between TRL 5 and 6.

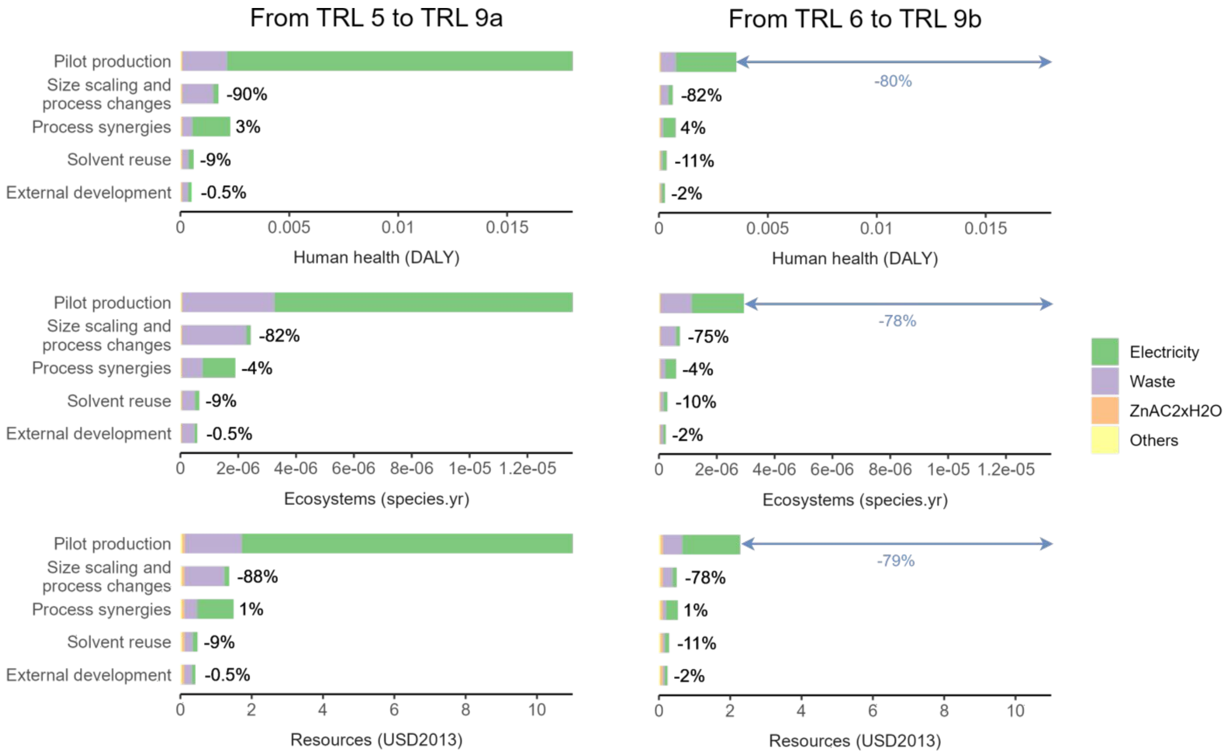

Figure S3. Endpoint impacts of TRLs 5, 6, 9a, and 9b, per kg of activator synthesized in the egalitarian perspective. Hotspots that contribute less than 10% to the endpoint impact were grouped under "Others". Pilot production was based on experimental data, and subsequent steps to industrial scale were predicted using pLCA for size scaling, process changes, process synergies, and solvent reuse. External developments were forecasted based on the solvent reuse scenario and for 2030 under IPCC SSP2-RCP2.6. Data labels show the change in impact relative to pilot production while the double-sided arrows show the relative difference between TRL 5 and 6.

Table S7. Endpoint impacts for of an average passenger car tire ("Baseline") and a tire made with the new activator ("Activator") for 2030 (IPCC SSP2-RCP2.6). The three ReCiPe perspectives are shown as well as best- and worst-case scenario for the tire made with the new activator. These results are shown in Figure 4 of the main body of the article.

Category	Unit	Perspective	Baseline	Activator	Activator worst case	Activator best case
Ecosystems	species.yr	Egalitarian	4.81E-05	4.35E-05	4.79E-05	3.91E-05
Ecosystems	species.yr	Hierarchist	5.98E-06	5.27E-06	5.93E-06	4.61E-06
Ecosystems	species.yr	Individualist	2.71E-06	2.39E-06	2.69E-06	2.10E-06
Human health	DALY	Egalitarian	4.75E-02	4.44E-02	4.75E-02	4.13E-02
Human health	DALY	Hierarchist	2.11E-03	1.89E-03	2.10E-03	1.68E-03
Human health	DALY	Individualist	3.04E-04	2.82E-04	3.03E-04	2.61E-04
Resources	USD2013	Egalitarian	1.70E+02	1.49E+02	1.68E+02	1.31E+02

Resources	USD2013	Hierarchist	1.70E+02	1.49E+02	1.68E+02	1.30E+02
Resources	USD2013	Individualist	1.69E+02	1.49E+02	1.68E+02	1.30E+02

3.2. Relative solvent reduction

A scenario analysis was conducted to quantify the effect of the relative solvent reduction during scale-up. This is a crucial parameter of our analysis because, while the water solvent itself does not have notable impacts, the treatment of the spent solvent is the main hotspot at high TRLs. In the baseline, a 20% relative reduction was applied as advised by Piccino et al. (2016). However, this lead to the prediction that TRL 9a would use 48kg of water per functional unit as input whereas it could empirically be seen at TRL 6 that much less water would be needed. The relative solvent reduction observed between TRL 5 and 6 is 67%. We modeled an industrial production of the activator based on TRL 5 data with the 67% relative solvent reduction instead of the baseline 20%.

Figure S2 shows the endpoint impacts of TRL 9a when an empirical value is used for the relative reduction in solvent during scale-up ("less solvent" scenario). By using a 67% value instead of the 20% baseline, the impacts of TRL 9a diminish by about 59% across endpoint categories. This is a result of the lower need for electricity, mainly for distillation (59% lower), but also for heating and stirring. The amount of waste to be treated also decreases by 66%. It can also be seen that TRL 9b has impacts about 21% lower across endpoint categories than TRL 9a with less solvent.

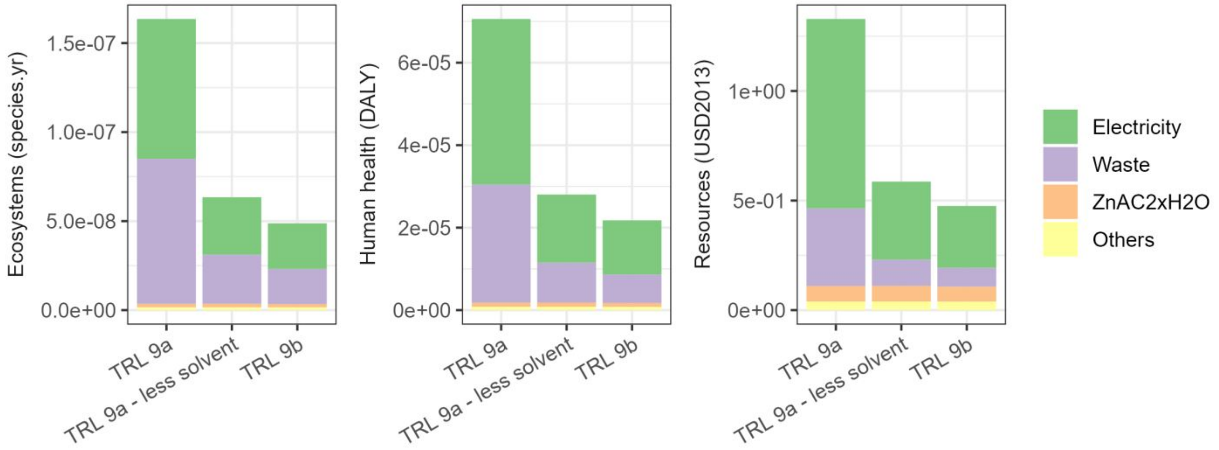

Figure S4. Endpoint impacts per functional unit of TRL 9a with less solvent as a result of a higher relative solvent recovery reduction. TRLs 9a and 9b are also shown for comparison. Hotspots that contribute less than 10% to the endpoint impact were grouped under "Others". All scenarios are shown with distillation, without external development, and in the hierarchist ReCiPe perspective.

References

1. Bittel, L. (2021). Synthesis and characterization of ZnO-NP@SiO2-NP with focus on upscaling. M.Sc. Dissertation, Fraunhofer ISC.
2. ESCO. (2022). *Laboratory Fume Hood*. https://escolifesciences.eu/products/download/Fumehood-Green-Ducted-Fumehood-Guide-Brochure-A4-LR.pdf (accessed 2023-08-10)
3. Fett, B. (2019). Evaluation of SiO2/ZnO nanoparticles for the reduction of ZnO in car tires. M.Sc. Dissertation, Fraunhofer ISC. http://urn.fi/URN:NBN:fi:aalto-201910275867 (accessed 2023-08-10)
4. Fisher Scientific. (2016). *Vacuum Pumps Vacuum Pumps for Lab & Industry Pump Selection and Application Guide*. https://assets.fishersci.com/TFS-Assets/CCG/EU/Welch-Vacuum-Technology/brochures/12139 FB Vacuum pumps_EN.pdf?_ga=2.248625479.1175172168.1691675961-522684238.1691675961 (accessed 2023-08-10)
5. Fisher Scientific. (2023). Fisherbrand Analytical Balances.
6. https://www.fishersci.co.uk/shop/products/analytical-precision-balances-10/p-7158705 (accessed 2023-08-10)
7. Fraunhofer ISC. (2020). *Progress of the set-up of the upscaling procedure of ZnO-NP@SiO2-NP synthesis*.
8. Fridrihsone, A., Romagnoli, F., Kirsanovs, V., & Cabulis, U. (2020). Life Cycle Assessment of vegetable oil based polyols for polyurethane production. *Journal of Cleaner Production*, *266*, 121403, DOI 10.1016/j.jclepro.2020.121403
9. Hennequin, T., Huijbregts, M. A. J., & van Zelm, R. (2022). The influence of consumer behavior on the environmental footprint of passenger car tires. *Journal of Industrial Ecology*, 1–14, DOI 10.1111/jiec.13334
10. Hughes, J. T., & Navrotsky, A. (2011). Enthalpy of formation of zinc acetate dihydrate. *The Journal of Chemical Thermodynamics*, *43*(6), 980–982, DOI 10.1016/j.jct.2011.02.004
11. Huijbregts, M. A. J., Steinmann, Z. J. N., Elshout, P. M. F., Stam, G., Verones, F., Vieira, M., Zijp, M., Hollander, A., & van Zelm, R. (2017). ReCiPe2016: a harmonised life cycle

impact assessment method at midpoint and endpoint level. *International Journal of Life Cycle Assessment*, *22*(2), 138–147, DOI 10.1007/s11367-016-1246-y

12. Kevin, T. (2020). Optimierung der Synthese von ZnO@SiO2-Partikeln für die Anwendung als multifunktionales Verstärkersystem in Kautschuken. M.Sc. Dissertation, Fraunhofer ISC.
13. Landi, D., Consolini, A., Germani, M., & Favi, C. (2019). Comparative life cycle assessment of electric and gas ovens in the Italian context: An environmental and technical evaluation. *Journal of Cleaner Production*, *221*, 189–201, DOI 10.1016/j.jclepro.2019.02.196
14. Larry R. Evans, MacIsaac Jr., J. D., Harris, J. R., Yates, K., Dudek, W., Holmes, J., Popio, J., Rice, D., & Salaani, M. K. (2009). NHTSA Tire Fuel Efficiency Consumer Information: Phase 2 - Effects of Tire Rolling Resistance Levels on Traction, Treadwear, and Vehicle Fuel Economy. National Highway Traffic Safety Administration, Springfield, Virginia. https://www-esv.nhtsa.dot.gov/Proceedings/22/files/22ESV-000303.pdf (accessed 2023-08-10)
15. Masanori Kan, A., Takuya Okazaki, T., & Tatsuo Sakashita, S. (1984). *United States Patent: Tire tread having low rolling resistance* (Patent No. 4,444,236).
16. Mehrkesh, A., & Karunanithi, A. T. (2013). Energetic ionic materials: How green are they? A comparative life cycle assessment study. *ACS Sustainable Chemistry and Engineering*, *1*(4), 448–455, DOI 10.1021/sc3001383
17. Merck. (2023). *Whatman Prepleated Qualitative Filter Paper*. https://www.sigmaaldrich.com/product/aldrich/wha1201150 (accessed 2023-08-10)
18. Muñoz, I., & Schmidt, J. H. (2016). Methane oxidation, biogenic carbon, and the IPCC's emission metrics. Proposal for a consistent greenhouse-gas accounting. *The International Journal of Life Cycle Assessment*, *21*(8), 1069-1075, DOI 10.1007/s11367-016-1091-z
19. Okel, Timothy A. (1998). Effect of silica on the viscoelastic properties of a model tread compound. *Rubber World*, *218*(3), 21–28.
20. Piccinno, F., Hischier, R., Seeger, S., & Som, C. (2016). From laboratory to industrial scale: a scale-up framework for chemical processes in life cycle assessment studies. *Journal of Cleaner Production*, *135*, 1085–1097, DOI 10.1016/j.jclepro.2016.06.164
21. Santos, P. G., Scherer, C. M., Fisch, A. G., & Rodrigues, M. A. S. (2020). Petrochemical wastewater treatment: Water recovery using membrane distillation. *Journal of Cleaner*

Production, *267*, 121985, DOI 10.1016/j.jclepro.2020.121985

22. Sacchi, R. (2023). premise_gwp. https://github.com/polca/premise_gwp (accessed 2024-02-04).
23. Susanna, A., Armelao, L., Callone, E., Dirè, S., D'Arienzo, M., Di Credico, B., Giannini, L., Hanel, T., Morazzoni, F., & Scotti, R. (2015). ZnO nanoparticles anchored to silica filler. A curing accelerator for isoprene rubber composites. *Chemical Engineering Journal*, *275*, 245–252, DOI 10.1016/j.cej.2015.04.017
24. Susanna, A., D'Arienzo, M., Di Credico, B., Giannini, L., Hanel, T., Grandori, R., Morazzoni, F., Mostoni, S., Santambrogio, C., & Scotti, R. (2017). Catalytic effect of ZnO anchored silica nanoparticles on rubber vulcanization and cross-link formation. *European Polymer Journal*, *93*, 63–74, DOI 10.1016/j.eurpolymj.2017.05.029
25. ThermoFisher Scientific. (n.d.). *Cimarec Stirring Hotplates Series*. 2023. https://www.thermofisher.com/order/catalog/product/SP88857108?SID=srch-srp-SP88857108#/SP88857108?SID=srch-srp-SP88857108 (accessed 2023-08-10)
26. Tullo, A. H. (2009). Stretching Tires' Magic Triangle. *Chemical & Engineering News*, *87*(46). https://cen.acs.org/articles/87/i46/Stretching-TiresMagic-Triangle.html (accessed 2023-08-10)
27. Vleugels, N., Pille-Wolf, W., Dierkes, W. K., & Noordermeer, J. W. M. (2015). Understanding the influence of oligomeric resins on traction and rolling resistance of silica-reinforced tire treads. *Rubber Chemistry and Technology*, *88*(1), 65–79, DOI 10.5254/rct.14.86947
28. Wernet, G., Bauer, C., Steubing, B., Reinhard, J., Moreno-Ruiz, E., & Weidema, B. (2016). The ecoinvent database version 3 (part I): overview and methodology. *The International Journal of Life Cycle Assessment*, *21*(9), 1218–1230, DOI 10.1007/s11367-016-1087-8
